# Analysis of operation procedure and effect for emergency surgery in general hospital during novel coronavirus pneumonia period

**DOI:** 10.1186/s12893-020-00852-2

**Published:** 2020-08-26

**Authors:** Yuchen Liu, Minggang Wang, Yingmo Shen, Jie Chen

**Affiliations:** Department of Hernia and Abdominal Wall Surgery, Beijing Chaoyang Hospital, Capital Medical University, Beijing, 100043 People’s Republic of China

**Keywords:** COVID-19, Pneumonia;hernia, Incarceration, Emergency operation

## Abstract

**Background:**

Novel coronavirus pneumonia (NCP) outbreak in Wuhan, China in early 2020, resulted in over 80 thousand infections in China. At present, NCP has an explosive growth in the world. Surgeons could refuse selective operation during the outbreak, but they must face the emergency operation. We hope to avoid the spread of NCP while ensuring efficient treatment of emergency cases.

**Methods:**

The data of patients with incarcerated hernia admitted to Beijing Chaoyang Hospital during NCP epidemic were analyzed and compared with those in 2019. All cases were divided into NCP group and 2019 group. The operation data and inpatient protection process of emergency cases were analyzed. Result During the NCP epidemic, 17 cases with incarcerated hernia were treated in our department. A Total of 263 cases of the same disease were admitted in 2019. There was no significant difference in age, gender, BMI and hernia type between two groups. No significant difference was observed between the two groups in operation method and hospital stay. The waiting time for emergency operation of NCP group was significantly longer than that of 2019 group (*P* = 0.002). A buffer ward was set up by administrator of hospital during NCP outbreak. Hospitals were divided into “Red area, Yellow area and Green area” artificially, and strict screening consultation system was implemented. There was no case of SARS-nCoV-2 infection in medical staff.

**Conclusion:**

It was safe and effective to carry out emergency operation on the premise of screening, protection and isolation during the NCP epidemic. The increased waiting time for operation due to NCP screening did not threaten medical safety of emergency incarcerated hernia patients.

## Background

**Fig. 1:**
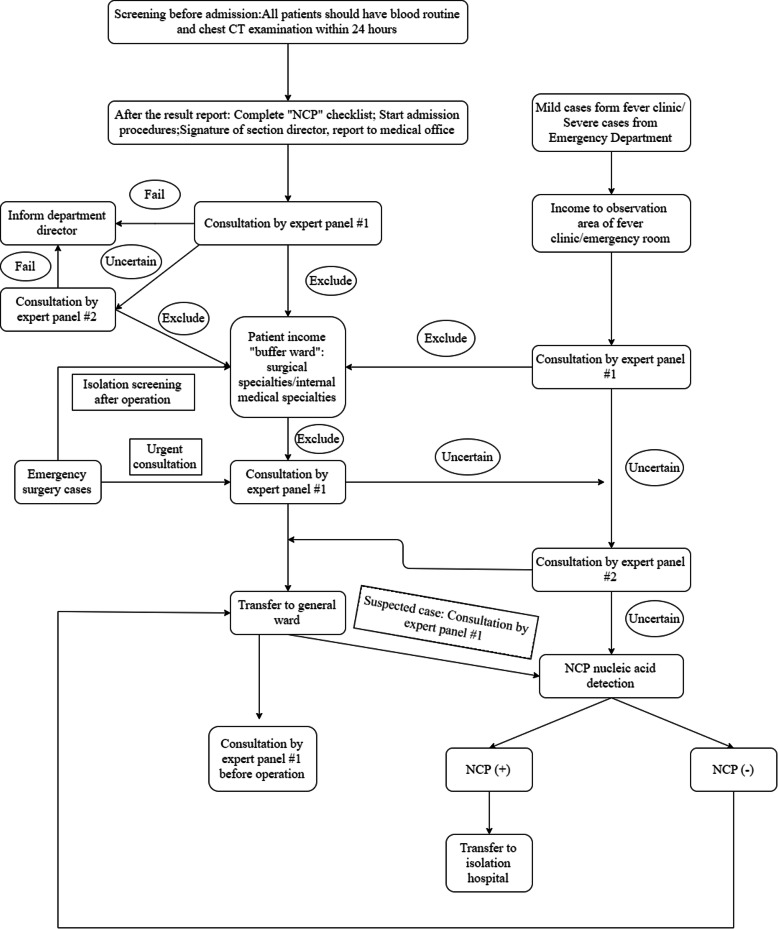
flow chart of admission process

**Fig. 2:**
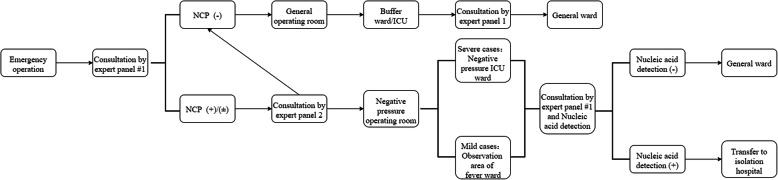
flow chart of surgical protection management

In December 2019, several cases of viral pneumonia occurred in Wuhan of China, and a large-scale outbreak occurred in China in a short period of time [1]. The novel coronavirus was named by WHO as 2019 new coronavirus (SARS-nCov-2), or novel coronavirus pneumonia (NCP) in January 2020 [2]. As of March 15th, 2020, more than 80,000 NCP cases have been confirmed, according to Chinese officials. But it was gratifying that the epidemic situation in China has been fundamentally improved. The number of confirmed cases decreased significantly, and most of them were imported cases. At present, NCP outbreaks have occurred in many countries and regions around the world [3]. Many countries began to use the Chinese model to block the development of the epidemic. During the outbreak, most of the surgical areas in China were vacant. All elective operations were suspended within the specified time, and only patients requiring emergency operations were admitted. Moreover, different regions had different treatment processes and policies, and different protective measures [4]. Most of hernia surgical diseases belong to selective operation [5]. Therefore, as a hernia specialist center, the treatment of elective patients was also stagnated in Beijing Chaoyang Hospital during the epidemic. However, the emergency treatment of incarcerated hernia was still in accordance with the emergency surgery [6], and relevant admission process (Fig.1) and surgical protection management (Fig.2) measures were formulated in order to avoid nosocomial infection, while solving emergency surgery, protect the life and health of patients and medical staffs. During the epidemic period, all patients with incarcerated hernia had no nosocomial infection after being admitted to hospital for operation, and the treatment effect was positive. The protective process and therapeutic effect of emergency hernia operation during NCP epidemic in Beijing Chaoyang Hospital was reported.

## Methods

### Study cohort

Beijing municipal government launched the first level response to major public health emergencies since January 24th, 2020. The data of incarcerated hernia patients were collected from January 24th to March 15th in Department of Hernia and Abdominal Wall Surgery, Beijing Chaoyang Hospital. Inclusion criteria: emergency incarcerated hernia patients who received emergency operation. At the same time, the data of the same disease cases of emergency surgery were collected in our department in 2019.

### Division and protection level of hospital

The hospital was divided into “Red area”, “Yellow area” and “Green area” according to the exposure risk level, and different levels of protection were carried out according to the exposure risk of different areas. The protection level was divided into four levels: (1) General protection: wear surgical masks (including staff and patients); (2) Level I protection: wear work clothes, work caps, surgical masks and gloves;(3) Level II protection: wear work clothes, isolation clothes, medical protective masks, shoe covers, work caps, goggles or protective masks; (4) Level III protection: on the basis of level II protection, wear comprehensive respiratory protective device.

“Red area” was defined as high-risk exposure area, including fever clinic, emergency department, operating center, department of laboratory, pathology department, intensive care unit and other work departments that may have direct contact with pathogens. The corresponding protection level of “Red area” was “Level III protection”. “Yellow area” was defined as buffer ward. After NCP screening, all emergency cases were admitted to the buffer ward. The ward was a separate floor in hospital, and only one emergency patient could be admitted to each room. The corresponding protection level of “Yellow Area” was “Level II protection”. “Green area” was defined as general surgical ward. Emergency patients could be transferred to general ward after expiration of isolation in the buffer ward, and only one emergency patient could be admitted to each room. The corresponding protection level of “Green Area” was “Level I protection” [7].

### Examination before admission

Admission examination included NCP screening and emergency operation related examination. All emergency patients were screened by blood test and pulmonary CT before admission and completed NCP screening form [8]. After being signed by the Department Director and consultation by the NCP expert panel (composed of respiratory department, radiology department, intensive care unit, infection department and emergency department), emergency cases could be admitted to the buffer ward. Preoperative preparations were carried out in the buffer ward.

### Surgical procedure

For patients with suspected NCP that cannot be excluded or with a history of close contact, the operation must be performed in a negative pressure operating room. After operation, they returned to fever observation area (Red Area) or negative pressure ward of ICU. For patients who had passed NCP screening, the operation could be performed in the general operating room. These patients returned to the buffer ward (Yellow Area). Open or laparoscopic surgery was selected according to the patient’s condition. And a corresponding level of protection according to exposure risk was performed [7].

### Post-operative cares

For the patients who return to the buffer ward after operation, blood test and pulmonary CT examination must be performed again after a week of isolation observation. The emergency patients in the buffer ward could only be transferred to general surgery ward (“Yellow area” to “Green area”) after consultation with the “experts panel” excluding NCP. During the postoperative treatment, patients with fever should be reported twice a day, and relevant screening should be carried out according to the opinions of the expert panel. Patients who had been isolated for less than one week but met the discharge standard could be discharged directly from the buffer ward.

### Determination of outcomes and follow-up

The general data of patients, waiting time for emergency operation, operation mode, operation time, intraoperative complications and postoperative complications were recorded. All emergency patients still needed to be followed up with NCP daily after discharge, such as body temperature, NCP symptoms, etc. [8]. In addition, surgical follow-up was carried out to understand recovery of operation. The follow-up time was 1 week, 1 month and 3 months after the operation [9].

### Statistical analysis

SPSS Statistics software (v 23.0) was used for data analysis. The results of descriptive analysis of continuous variables are expressed as mean ± SD and were calculated using the independent samples t-test. Categorical variables are presented as number or percentage and were calculated using the χ2 test. *P* < 0.05 was considered to denote statistical significance.

## Results

From January 24th to March 15th, data of 17 emergency patients were included in this study (Tab.1). There were 9 males and 8 females, including 9 cases of incarcerated inguinal hernia, 4 cases of incarcerated umbilical hernia, 2 cases of incarcerated incisional hernia, 1 case of incarcerated parastomal hernia and 1 case of incarcerated obturator hernia. At the same time, 263 cases of emergency patients with same disease in 2019 were included, including 199 cases of incarcerated inguinal hernia, 31 cases of incarcerated umbilical hernia, 23 cases of incarcerated incisional hernia, 8 cases of incarcerated parastomal hernia and 2 cases of incarcerated obturator hernia. The age, BMI and hospital stay in NCP group and 2019 group were 68.17 ± 9.47y vs. 62.74 ± 11.25y, 22.85 ± 4.24 kg/m^2^ vs. 23.41 ± 4.33 kg/m^2^ and 7.52 ± 3.93d vs. 7.98 ± 4.41d, respectively. There was no significant difference in gender, age, BMI and type of incarcerated hernia between two patients (*P* > 0.05). The waiting time of emergency operation in the two groups was 5.53 ± 0.73 h vs. 2.78 ± 0.33 h with statistically significant difference(*P* = 0.002). In NCP group, laparoscopic surgery was performed in 2 cases and open surgery in 15 cases. Suture was performed in 6 cases and mesh repair in 11 cases. There were 2 cases of hernia necrosis. In 2019 group, 88 cases underwent laparoscopic surgery, 175 cases underwent open surgery. The difference was statistically significant (*P* = 0.049). In NCP group, 7 cases were transferred to general surgery ward through buffer ward, and 10 patients were discharged directly from buffer ward. Fever occurred in 2 patients with NCP screening by expert panel consultation post-operation. Follow up of novel coronavirus pneumonia: NCP was excluded from 13 patients after two weeks follow-up and surgical follow-up continued; No NCP was observed in 4 cases during 1 week follow-up and observation continued. Surgical follow-up: No intraoperative complications occurred; there was no infection, recurrence or intestinal obstruction in the short-term follow-up (Tab. 2).

**Table 1:** Details of 17 emergency cases during NCP

No.	Gender	Age range (y)	BMI (kg/m^2^)	Waiting time for the intervention (h)	Hospital Stay (d)	Primary diagnosis	Operative time (Min)	Anesthesia	Repair method	Discharge Ward	ICU Ward	Remark
1	1	50–59	27.68	6	4	GH	110	Iv	Lichtenstein	IW	N	
2	2	70–79	17.33	5.5	6	GH	45	LA	Plug	IW	N	
3	2	70–79	20.70	5	4	GH	35	GA	TAPP	IW	N	
4	1	60–69	21.49	6.5	2	UH	55	Iv	Open Suture	IW	N	
5	1	70–79	24.91	5.5	13	IH	62	GA	Onlay	SW	N	
6	1	60–69	17.96	6.5	14	GH	265	GA	Open Suture	SW	N	bowel necrosis
7	1	60–69	24.77	5.5	6	GH	100	LA	Lichtenstein	IW	N	
8	2	60–69	18.90	4.5	13	UH	65	Iv	Open Suture	SW	N	
9	2	60–69	27.89	4.5	4	GH	65	Iv	Lichtenstein	IW	N	
10	1	70–79	18.94	5	4	GH	65	Iv	Plug	IW	N	
11	1	70–79	23.66	6.5	13	PH	113	GA	Open Suture	SW	N	
12	2	80–89	28.30	5.5	10	IH	95	GA	Sublay	SW	N	
13	2	70–79	28.91	4.5	6	UH	80	GA	Onlay	IW	N	
14	1	50–59	27.55	6	4	GH	60	Iv	Lichtenstein	IW	N	
15	1	70–79	18.72	6	10	GH	70	Iv	Lichtenstein	SW	N	
16	2	60–69	23.73	6.5	6	UH	80	LA	Open Suture	IW	N	bowel necrosis
17	2	80–89	17.07	4.5	9	OH	120	GA	Lap Suture	SW	Y	

GH = Groin hernia, UH=Umbilical hernia, IH=Incisional hernia, PH=Parastomal hernia, OH = Obturator hernia, Iv = intravenous anesthesia,

LA = local anaesthesia, GA = general anesthesia, IW = Isolation ward, SW = Surgical ward

**Table 2:** Data of NCP group and 2019 group

Characteristic	Measure	Emergency Cases during NCP	Emergency Cases during 2019	t/χ2	*P* value
Age	Mean (SD), y	68.17 (9.47)	62.74(11.25)	−0.091	0.891
Sex				1.232*	0.298
	Male, No. (%)	9 (52.94)	174(66.16)		
	Female, No. (%)	8 (47.06)	89(33.84)		
BMI	Mean (SD), Kg/m^2^	22.85(4.24)	23.41 ± 4.33	−4.567	0.737
Hernia Type				5.343	0.198
	Inguinal hernia No. (%)	9(52.94)	199(75.67)		
	Incisional hernia No. (%)	2 (11.77)	23(8.75)		
	Umbilical hernia, No. (%)	4(23.53)	31(11.79)		
	Parastomal hernia, No. (%)	1(5.88)	8(3.04)		
	Obturator hernia, No. (%)	1(5.88)	2(0.76)		
Operation Method				3.446*	0.049
	Open, No. (%)	15 (88.24)	175(66.54)		
	Laparoscopy, No. (%)	2(11.76)	88(33.46)		
Repair Method				3.969*	0.046
	Suture, No. (%)	6(35.29)	43(16.35)		
	Mesh, No. (%)	11(64.71)	220(83.65)		
Discharge Mode				–	–
	Isolation ward, No. (%)	7(41.18)	–		
	Surgical ward, No. (%)	10 (58.82)	–		
Waiting time for the intervention	Mean (SD), h	5.53 (0.73)	2.78 (0.33)	3.021	0.002
Hospital stay	Mean (SD), d	7.52 (3.93)	7.98(4.41)	1.746	0.654

*: χ2 test

## Discussion

Hernia and abdominal wall surgery are the branches of general surgery, and types of diseases are relatively concentrated. Most of the operations are elective, including some limited time operations and emergency operations [10]. During the NCP epidemic, diagnosis and treatment should be classified according to the condition. Our department had stopped the treatment of all patients undergoing elective surgery since the first level response was launched in Beijing. At the same time, confine operation should be postponed, but emergency operation was still needed. The emergency operation of hernia surgery is mainly incarcerated inguinal hernia, incarcerated umbilical hernia, incarcerated incisional hernia, incarcerated parastomal hernia and so on [5]. In order to save medical resources and reduce the exposure of medical staff and patients, we could suspend the treatment of patients undergoing elective surgery as a special department for hernia and abdominal wall surgery. But for incarcerated hernia emergency patients, we could only choose active surgical intervention [11]. For such cases, NCP guidelines should be done in accordance with the protection requirements of hospital and isolation to avoid hospital infection. At the same time, effective protection of medical staff and other patients could be carried out in the treatment of incarcerated hernia cases [7].

In terms of choice of surgical methods for emergency incarcerated hernia cases, we were faced with elderly patients, many basic diseases, long history or other characteristics. Many of these patients had a high risk of general anesthesia. Therefore, many emergency patients in our department chose open surgery under local anesthesia during NCP epidemic [12]. According to previous limited experience, SARS-CoV-2, which was mainly transmitted by droplets, was similar to the Middle East respiratory syndrome (MERS) coronavirus found in September 2012 [13]. Seddiq reported the cases of MERS after CABG in 2017, without special protection during operation. No infection was found in 40 close contacts [14]. Some hospitals in South Korea performed emergency operations for 4 suspected cases and 2 confirmed cases of MERS under strict protection [15]. No staff infection was found. During the outbreak, a hospital in Wuhan performed cesarean section for 48 pregnant women suspected and confirmed NCP, and no infection was found among the medical staff. SARS-CoV-2 was similar to MERS coronavirus and could be transmitted through droplets and feces [16]. In contrast, SARS-CoV-2 may even spread through aerosols. Considering the management of airway by general anesthesia intubation and the possibility of aerosol diffusion in laparoscopic surgery, most of our surgical methods were open surgery in NCP epidemic [17]. The main anesthesia was local anesthesia combined with intravenous anesthesia in order to avoid the spread of pathogens, by cutting off route of transmission. Of course, there were also cases that were actually more suitable for laparoscopic surgery, such as incarcerated obturator hernia [18], which could also be performed under premise of good protection. However, the operating room should be regarded as “Red Area” and the corresponding high-level protection should be performed. During the epidemic period, proportion of laparoscopic surgery was 11.76%, and proportion of previous emergency laparoscopic surgery was 33.46%, although the difference was not statistically significant. But we preferred open surgery during NCP outbreak.

During NCP epidemic, treatment process of emergency incarcerated hernia patients was more complicated than ever, and more examinations and screening work had been carried out. Because in the process of admission, once it caused exposure or infection, it was fatal for the whole hospital. Although we had increased admission process, resulting in a significant increase in emergency surgery waiting time compared to cases in 2019, we believed it worthy during NCP outbreak. Moreover, compared with the previous emergency patients, strangulation of incarcerated hernia did not increase significantly. So we had reason to think that strict pre hospital screening process was necessary, including blood test and pulmonary CT. Of course, contact history of epidemic areas and family members was also essential [19]. All the relevant examination results needed to be consulted by expert panel before entering the hospitalization process. The expert panel must be composed of specialists with rich experience. In our opinion, the expert group should be composed of senior doctors from respiratory department, infection department, radiology department, emergency department and intensive care unit. At least two groups of experts shall be equipped to ensure 24-h standby status. However, after the operation, it was not allowed to enter the “Green area”. It was necessary to return to the “Yellow Area” for at least one week of isolation observation according to the condition. If possible, we even suggested that the observation isolation time extended as much as possible. In the “Yellow Area”, it is not allowed to visit or stay with family members. Since the incubation period of NCP was about 1 ~ 14 days, mostly 3 ~ 7 days [20], we chose to take the new NCP assessment when patients had been isolated for 7 days. In the early stage of the epidemic, patients in “yellow area” did not carry out nucleic acid monitoring 7 days after isolation instead of blood test and pulmonary CT examination. After consulting by expert panel again, patient could be transferred from buffer ward to general surgery ward (“Yellow to Green”). The rest of treatment continued in the general ward. In the Green area, a fixed family member was allowed to stay with patient, and the family member was not allowed to leave the ward until patient discharged. At present, we suggest that NCP test kit should be included in the routine screening examination, so as to ensure the accuracy of the examination. Discharge did not mean the end of isolation of patient. We still require patients to be isolated at home after discharge and did a good job of follow-up investigation. After NCP was excluded during the incubation period, surgical follow-up was continued. If NCP was suspected or diagnosed, the patients performed emergency surgery should be sent to Red area for treatment. Including the family members of the patients, nobody was allowed to enter Yellow or Green areas. It was better to transfer to a special designated hospital [7].

Compared with same emergency incarcerated hernia cases in 2019, hospital stay did not increase, and there was no significant difference in repair methods in NCP group. Therefore, we believed that it was safe to use mesh for emergency surgery during the epidemic. In addition, special protection did not increase the incidence of intraoperative complications. High level of protection was necessary for intraoperative protection, especially for suspected and confirmed cases. On the other hand, the emergency incarcerated hernia surgery under the epidemic situation should choose the simplest way of operation. The operation method that could achieve therapeutic effect in the shortest time was the one that should be selected during NCP epidemic [5].

So far, the number of newly confirmed NCP cases in China has declined rapidly. There have been no newly confirmed cases in Wuhan for two consecutive days, and most of confirmed cases are imported ones in China. The NCP epidemic prevention and control in China has achieved phased results. We hope to summarize the experience of emergency operation during NCP epidemic and achieve the highest efficiency of treatment and the safest protection in the face of similar situations. Therefore, the number of emergency surgery cases in our hospital was not enough, and data included in the study was not enough during NCP epidemic period. Secondly, the follow-up time of surgical patients was very short, which did not reached long-term follow-up, so we need to continue to observe. Thirdly, admission process and operation process were just our experience summary. Beijing was not the area with the most severe NCP epidemic, and the infection density might be smaller than that of Wuhan.

## Conclusion

It was safe and effective to carry out emergency operation on the premise of screening, protection and isolation during the NCP epidemic. The increased waiting time for operation due to NCP screening did not threaten medical safety of emergency incarcerated hernia patients.

## Data Availability

The datasets used and/or analysed during the current study are available from the corresponding author on reasonable request. The datasets used and/or analysed during the current study are available from the Department of hernia and abdominal wall surgery, Beijing Chaoyang Hospital, Capital Medical University, on reasonable request.

## Abbreviations

NCP: Novel Coronavirus Pneumonia
BMI: Body Mass Index
WHO: World Health Organization
MERS: Middle East Respiratory Syndrome
